# Highly Sensitive Room-Temperature Sensor Based on Nanostructured K_2_W_7_O_22_ for Application in the Non-Invasive Diagnosis of Diabetes

**DOI:** 10.3390/s18113703

**Published:** 2018-10-31

**Authors:** Md Razuan Hossain, Qifeng Zhang, Michael Johnson, Danling Wang

**Affiliations:** 1 Department of Electrical and Computer Engineering, North Dakota State University, Fargo, ND 58102, USA; mdrazuan.hossain@ndsu.edu (M.R.H.); qifeng.zhang@ndsu.edu (Q.Z.); 2 Materials and Nanotechnology Program, North Dakota State University, Fargo, ND 58102, USA; michael.johnson.1@ndsu.edu

**Keywords:** diabetes, acetone, biomarker, non-invasive, blood glucose, nanostructured K_2_W_7_O_22_, ferroelectric property, volatile organic compound

## Abstract

Diabetes is one of the most rapidly-growing chronic diseases in the world. Acetone, a volatile organic compound in exhaled breath, shows a positive correlation with blood glucose and has proven to be a biomarker for type-1 diabetes. Measuring the level of acetone in exhaled breath can provide a non-invasive, low risk of infection, low cost, and convenient way to monitor the health condition of diabetics. There has been continuous demand for the improvement of this non-invasive, sensitive sensor system to provide a fast and real-time electronic readout of blood glucose levels. A novel nanostructured K_2_W_7_O_22_ has been recently used to test acetone with concentration from 0 parts-per-million (ppm) to 50 ppm at room temperature. The results revealed that a K_2_W_7_O_22_ sensor shows a sensitive response to acetone, but the detection limit is not ideal due to the limitations of the detection system of the device. In this paper, we report a K_2_W_7_O_22_ sensor with an improved sensitivity and detection limit by using an optimized circuit to minimize the electronic noise and increase the signal to noise ratio for the purpose of weak signal detection while the concentration of acetone is very low.

## 1. Introduction

Diabetes, the seventh leading cause of death in the United States, is a precursor to a heterogeneous group of disorders and is indicated by high blood glucose levels [1,2]. Based 2017 statistics, about 30.3 million people in the U.S. have diabetes, or about 9.4% of the U.S. population [3]. There are many ways to diagnose type 1 diabetes [4], such as the A1C test (a blood test that indicates blood glucose levels) [5], fasting plasma glucose test (FPG), or oral glucose tolerance test (OGTT) [6]. However, these diagnosing methods are inconvenient, costly, and painful. Also, if we can have a device which can be a tool to screen or diagnose diabetes at a very early stage, it can particularly prevent the population with prediabetes to develop full-scale diabetes.

Volatile organic compounds (VOCs) [7] in human breath, identified in the early 1970’s [8], act as noninvasive indicators of human health. Some VOCs are known biomarkers for specific human diseases [9]. For instance, toluene can be used to trace lung cancer [10] and acetone can be used to monitor blood glucose level for diabetes [11,12]. The advantages of using VOCs as diagnostic tools include being harmless to the body, convenient to carry, low cost, and non-invasive. If a VOC can be used as a biomarker for diabetes mellitus and becomes a method to diagnose disease at an early stage with a convenient, much cheaper, and non-invasive way, most patients could be benefit from this technique and get their diseases controlled in time. In fact, researchers have proven that in general there is a positive correlation between blood glucose and breath acetone [13,14,15,16,17,18,19,20,21,22,23]. Therefore, it is reasonable to develop a device for the detection of breath acetone as an effective tool of diabetes diagnosis and screening.

There are many ways to detect acetone from exhaled breath, such as: gas chromatography-mass spectrometry (GC-MS) [24,25]; selected ion flow tube mass spectrometry (SIFT-MS) [26]; proton transfer reaction-mass spectrometry (PTR-MS) [27]; high-performance liquid chromatography (HPLC) [28]; ion mobility spectrometry (IMS) [29,30]; laser spectroscopic techniques, including tunable diode laser absorption spectroscopy (TDLAS) [31], and cavity ringdown spectroscopy (CRDS) [32]. These techniques are complicated in operation, expensive, and unavailable in small clinic or home settings [33]. In addition to techniques for acetone detection, there are many materials developed to detect acetone in various sensor devices, such as Pt-InN [34], Polypyrrole (PPy)-WO_3_ [35], Ni/InGaN/GaN [36], and Pd/TiO_2_/Si [37]. Compared to these materials, nanostructured K_2_W_7_O_22_ (KWO) shows the highest sensitivity to acetone even at room temperature. Our recent investigation of the sensing mechanism of KWO for acetone detection reveals that excellent room-temperature ferroelectric properties and porous nanostructure of KWO provide an effective chemiresistive reaction between high polar acetone and KWO [38]. This makes KWO a promising material to detect acetone for the application of non-invasive diabetes diagnosis.

Generally, in exhaled breath, the acetone concentration is usually in the range of 0.3–0.9 ppm (parts per million) for a healthy person and above 1.8 ppm for a diabetic patient [13]. People with breath acetone concentration between 0.9 ppm and 1.8 ppm can be considered prediabetes, who have a high risk to become diabetes. The parts-per-million concentration is just in a trace level, which is indeed quite challenge to be sensitively detected. Therefore, increasing the sensitivity and detection limit is the key while designing a sensor device to detect trace level acetone in the purpose of diabetes diagnosis. Usually, there are two major factors that can affect the sensor performance: one is electrical circuits for signal collection; the other is the sensing material properties. The preliminary results exhibited that KWO can sensitively detect acetone with a good detection limit around 1.2 ppm [17,38]. While considering its potential application in diabetes, such a detection limit of ≈1.2 ppm is not good enough to detect the breath acetone level in many people who are pre-diabetic. To solve the issue of weak signal detection and improve the sensor detection limit, we designed and modified the circuit system of sensor detection by: (1) introducing an operational amplifier (op-amp) [39] in the circuit to magnify the weak signal, and (2) employed a Wheatstone bridge [40] technique to balance the circuit and accurately measure the change of resistance caused by the interaction between KWO and trace level acetone.

In this paper, we report a breath sensor based on a novel nanostructured material, K_2_W_7_O_22_, to work as a chemiresistive sensor for the detection of acetone at room temperature. An advanced circuit system has been developed and described in detail regarding the improvement of the signal to noise ratio and the weak signal pick-up while considering the concentration of breath acetone is very low in the range of parts-per-billion (ppb) to parts-per-million (ppm). The sensing performance based on the improved circuits has been presented.

## 2. Circuit Design

**Figure 1:**
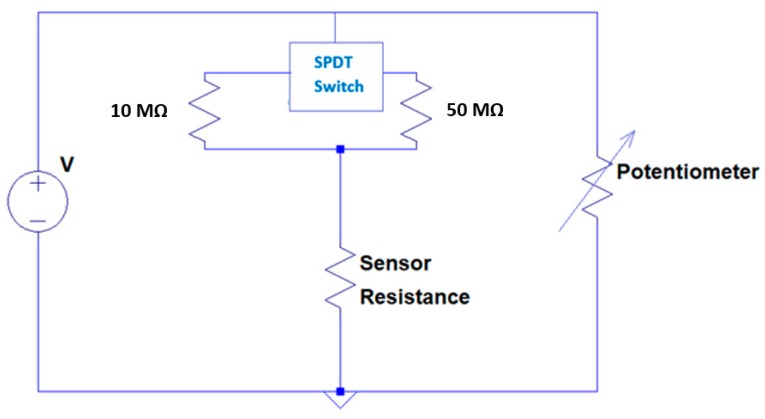
Schematic diagram of Wheatstone bridge.

**Figure 2:**
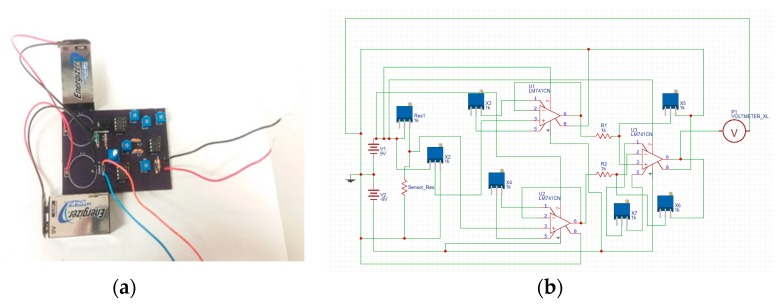
(**a**) PCB layout of the gas detection circuit; (**b**) Schematic diagram of the amplified circuit.

To detect the signal, we made a cost-efficient circuit with components such as resistors, potentiometers, op-amp (model name: LM741 CNNS) [41], and 9 V battery. The printed circuit board (PCB) was designed by OSHPARK (a PCB fabrication company, Portland, OR, USA) [42]. As shown in Figure 1, we used 10 MΩ and 50 MΩ resistors connected with a single pole double through (SPDT) switch [43] for compatible adjustment with the sensor resistance while testing different ranges of acetone concentration. A 10 MΩ resistor was used to calibrate the sensing response for lower concentrations of acetone from 0 to 6.25 ppm and a 50 MΩ resistor was chosen to calibrate the sensing response for a broad range of acetone from 0–50 ppm. The other branch of the Wheatstone bridge was introduced with a potentiometer to make zero correction of the circuit. Also, we introduced buffer amplifiers [44] in the circuit to avoid the impedance problem and to get unity gain. Because the signal from sensor detection is weak, we used a differential amplifier to amplify the signal. The voltages were taken from the two branches of the Wheatstone bridge. The amplification ratio was set up in accordance with the range of acetone detection. For instance, to detect a low concentration of acetone (0–6.25) ppm, we needed to amplify the signal 10-fold. Figure 2a shows our PCB circuit board and Figure 2b shows the schematic diagram of the circuit. We used two 9 V batteries to make +9 V and −9 V potential differences. The amplification of the signal was set in between the range of −9 V to +9 V and the output was clipped beyond that range. The output from the circuit was measured on an electrometer.

## 3. Experimental Setup

**Figure 3:**
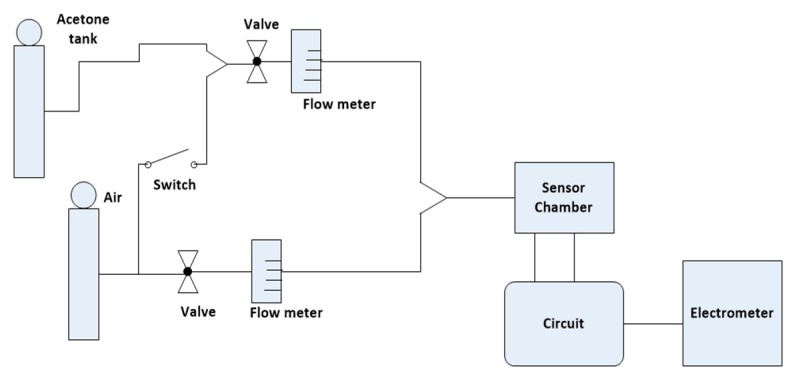
Block diagram of the testing system.

We tested the sensor with 0–6.25 ppm and 0–50 ppm concentrations of acetone. Our primary goal was to check the sensitivity of the KWO sensor to acetone with the improved circuit while the concentration of acetone was low (0–6.25 ppm). The other goal was to make sure that the circuit could functionally work nicely in a broad range of acetone concentration from 0 to 50 ppm. Figure 3 shows the sketch of the whole testing system. Acetone gas, supplied from the tank, was at the concentration of 50 ppm. The concentration of acetone could be diluted via mixing 50 ppm acetone with air and quantified through mass flow controllers. The sensor was put in a chamber and connected to the circuit for signal detection and collection. The circuit output was monitored from the electrometer and collected via computers.

## 4. Sensing Mechanism

**Figure 4:**
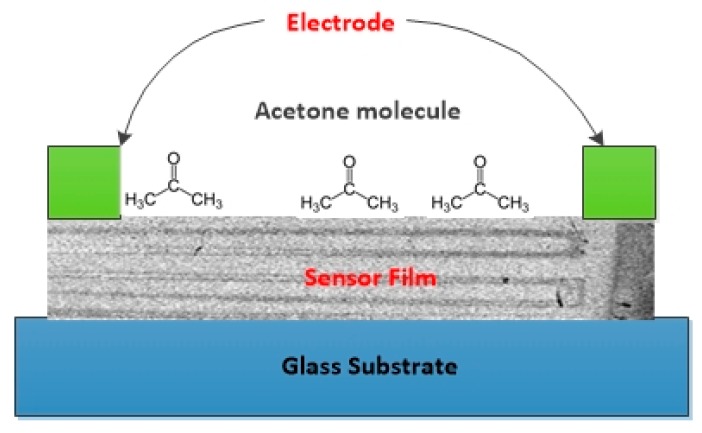
Electrostatic attraction between acetone molecules and the KWO sensor.

In the earlier section, we mentioned that the KWO sensor is sensitive to the acetone gas. Figure 4 shows the sensing interaction between the acetone molecule and the KWO sensor. Nanostructured KWO was measured to be a p-type semiconductor via a Hall effect measurement (Ecopia HMS-3000). Also, it showed good room-temperature ferroelectric properties [38]. All these unique properties of KWO can make it effectively attract high polar acetone molecules and result in an increase of the resistance [38,45,46]. Therefore, a KWO sensor can also be called chemiresistive sensor.

Theoretically, chemiresistive-based sensors can detect even tiny amounts of change of charges, e.g., electrons or holes. This can be reflected via a small change of resistance of sensing material, e.g., KWO, when it is exposed to a very low concentration of acetone. Generally, due to the limitations of the detecting circuit, device structure, etc., it is not easy to observe such a small change of resistance while the acetone concentration is too low. Considering the practical application of KWO sensor in diabetes, it is necessary to sensitively detect a concentration of acetone less than 0.9 ppm. The circuit mentioned above was designed to optimize the signal to noise ratio and realize a weak signal detection while a tiny resistance change is introduced by a small amount of acetone.

## 5. Results and Discussion

Sensitivity is the most important parameters for evaluating the sensing performance of sensors [47]. Sensitivity is defined as the variation in current ratio for specific gas concentration. If I_gas_ and I_air_ are the current values of the sensor, then the sensitivity, S [34] is:

$$\mathrm{Sensitivity}\;(S)=\frac{I_{\mathrm{gas}}-I_{\mathrm{air}}}{I_{\mathrm{air}}}$$

Sensitivity can be also measured in terms of voltage [48] and resistance [49].

**Figure 5:**
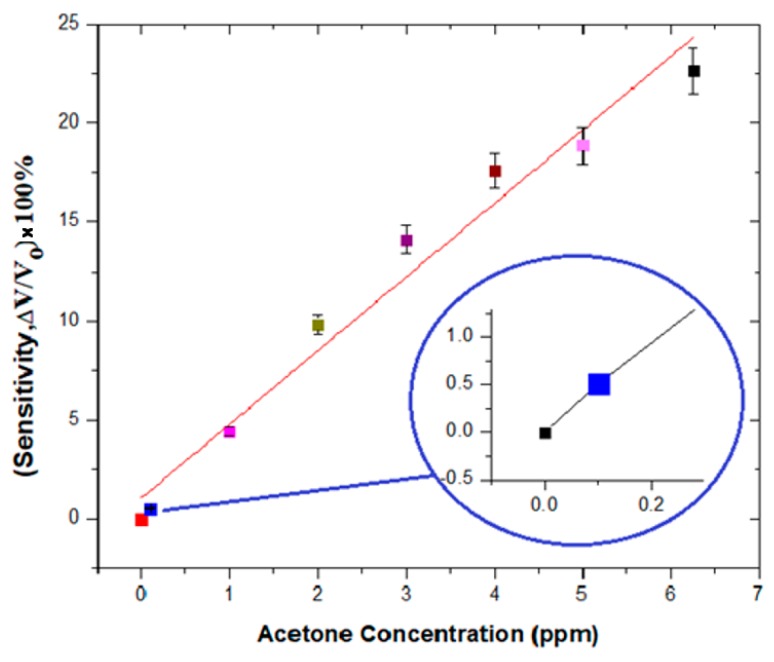
Sensitivity readings due to change in concentration of acetone for 0–6.25 ppm.

We collected two sets of data from our testing system. Figure 5 shows the sensitivity in terms of voltage that was found for the acetone concentration from 0 to 6.25 ppm. From the curve, we see that the sensitivity shows a linear relationship between the detected signal to the acetone from 0 to 6.25 ppm. Also, Figure 5 reveals an improvement of the sensitivity of KWO sensor when detecting acetone. The sensitivity was about 50.75% even when the concentration of acetone was only about 0.1 ppm.

We compared the sensing performance of the KWO sensor to low concentrations of acetone, 0–6.25 ppm, with and without using improved circuits as the signal collection. Table 1 shows the detection limit and sensitivity while the KWO sensor system employed the optimized detecting circuit. For example, the sensitivity of 1.0 ppm of acetone with the improved circuit was 441.1%, while the sensitivity without the improved circuit at 1.0 ppm of acetone was only 10%. The results indicated that the improved circuit significantly improved the sensitivity and detection limit of the KWO sensor. This is a very important improvement, in particular, considering the KWO sensor in application for the purpose of early stage type-1 diabetes diagnosis.

**Figure 6:**
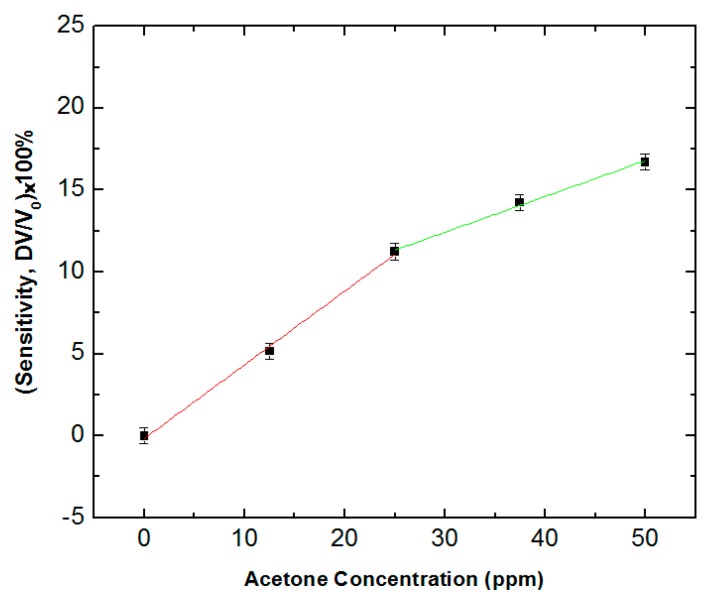
Sensitivity readings due to changes in concentration of acetone for 0–50 ppm.

We also measured the voltage change for the acetone concentration from 0 to 50 ppm. Figure 6 shows the sensitivity for the acetone concentration from 0 to 50 ppm. The results indicated a quite linear relationship between the sensitivity and the concentration of acetone from 0 to 25 ppm for the KWO sensor to detect acetone. However, when the concentration of acetone was higher than 25 ppm, the increase of sensitivity was a little bit offset with the increase of acetone concentration. This was because the resistance of the KWO sensor was higher than 50 MΩ while it was exposed with a higher concentration of acetone such as more than 25 ppm. Such a high resistance made the output voltage from the voltage divider branch unable to make a proportional change for the corresponding acetone concentration. Therefore, it was difficult for the circuit to show a broader linear response with the change of acetone concentration from 0 to 50 ppm. This is one limitation of this improved circuit, which we need to further optimize in order to make this circuit be compatible with a broad range of acetone concentrations.

## 6. Conclusions

To conclude, a modified signal detection system with new circuits was specifically designed for weak signal detection. The results show a significant improvement of the sensitivity of the KWO sensor device has been reached, which is very helpful for early stage detection of diabetic. For future work, our plan is to develop a circuit that can read the value linearly with the change of acetone concentration from 0 to 50 ppm or even higher (good dynamic range). Also, we will try to digitize the relationship between this voltage readings and the blood glucose level. Therefore, the patient can read their blood glucose level directly from the readings. Furthermore, we will plan to use the as-designed breath sensor to do in vivo breath testing from patients.

## Figures and Tables

**Table 1:** Sensitivity comparison between improved circuit measurement system (sample 1) and previous resistance measurement system (sample 2) for acetone concentrations of 0–6.25 ppm.

Acetone Concentration (ppm)	Sample 1 (Sensitivity, ΔV/Vo) × 100%	Sample 2 (Sensitivity, ΔR/Ro) × 100%
0	0	0
1	4.411	0.1
2	9.823	0.2
3	14.117	0.225
4	17.588	0.245
5	18.882	0.28
6.25	22.647	0.29
